# Modern forensic psychiatric hospital design: clinical, legal and structural aspects

**DOI:** 10.1186/s13033-018-0238-7

**Published:** 2018-10-20

**Authors:** Allan Seppänen, Iida Törmänen, Christopher Shaw, Harry Kennedy

**Affiliations:** 1 Department of Psychoses and Forensic Psychiatry, Helsinki University Hospital, Helsinki, Finland; 2 Vanha Vaasa Hospital, Vaasa, Finland; 3 Department of ICT Psychiatry and Psychosocial Treatments, Helsinki University Hospital, Helsinki, Finland; 4 Medical Architecture and Art Projects Ltd, London, UK; 5 Department of Psychiatry, Trinity College, Dublin, Ireland; 6 National Forensic Mental Health Service, Central Mental Hospital, Dundrum, Dublin, Ireland

**Keywords:** Forensic psychiatry, Hospital architecture, Service development

## Abstract

Forensic psychiatric care must be provided within the least restrictive setting possible, whilst simultaneously maintaining appropriate levels of security. This presents particular challenges for the design of forensic psychiatric hospitals, which are required to provide both a therapeutic and a safe material environment, often for extended periods of treatment and rehabilitation. By taking into consideration variable trends in psychiatric service provision and myriad clinical, legal and ethical issues, interdisciplinary forensic facility design teams are at the very forefront in implementing the latest developments in medical architecture. Also, although there are significant differences in how forensic psychiatric services are organized around the world, the underlying clinical challenges and increasingly research-based treatment principles are similar worldwide; it is therefore becoming less acceptable to operate and develop national forensic services without reference to international standards. Accordingly, we here review the literature on what features of forensic psychiatric facilities best serve the needs of those patients who need to rely on them, and we present a systematic and widely applicable approach to the complex and costly challenge of modern forensic psychiatric hospital design.

## Background

Forensic psychiatric care aims to improve the mental health and reduce the risk of recidivism of mentally disordered offenders, and other patients with similar complex needs. This must be achieved within the least restrictive setting possible, and with a view to eventual community reintegration, whilst simultaneously maintaining a secure treatment environment [1–3]. However, forensic psychiatric services and the facilities in which they are provided are defined and governed in different ways across the world: some countries have issued detailed criteria for different levels of secure care, including building design and material specifications [4–6], whereas in other countries, security is much more loosely defined, and has essentially developed over time along with clinical practices [7–9]. This is mainly due to variation in the specifics of legislation and the particular ways in which forensic mental health systems are operationalized in different countries, as the underlying clinical issues and principles remain relatively similar across countries [10–13].

Accordingly, the placement and treatment of the forensic patient population has been an issue of debate within the criminal justice systems in Western Europe [14, 15]. In addition to dedicated forensic hospitals and units, mentally disordered offenders may be placed in general psychiatric hospitals, prison hospitals and, less commonly, psychiatric wards in general hospitals. In some EU member states, not only mentally disordered offenders, but other aggressive, violent or otherwise high-risk patients referred from general psychiatric facilities may be admitted to forensic facilities [12, 14]. This can cause strain between services, as general psychiatry may develop a tendency towards outsourcing to forensic psychiatry the treatment—particularly coercive interventions—of patients viewed as somehow troublesome [16] and, on the other hand, forensic patients may be seen to block beds needed for patients with more acute presentations [17]. In any case, interaction between general psychiatric services and forensic psychiatry is essential, and inevitable: the majority of patients in forensic units have had previous contact with general psychiatric services and will require transfer back to communal care at some point in their psychiatric treatment [18].

Deinstitutionalization, particularly in Western Europe and the United States, has gradually reformed psychiatric health care; many large, institution-like hospitals have been withdrawn from use or modified since the 1950s [19] and many units face continual pressure to decrease the number of remaining beds. The trend is being reinforced by developments in information technology, which are continually offering new treatment possibilities and environments for psychiatric care [20]. Arguably, the trend towards deinstitutionalization of general psychiatric patients has contributed to increasing the number of placements in forensic psychiatric facilities and raising the demand for supported housing, in a process of re- or trans-institutionalization [17, 19, 21, 22]. This is not a new area of enquiry; the relationship between the decrease in general psychiatric bed numbers and the expansion of the prison population suffering from mental health issues has been a focus of intense research and debate since the 1930’s, when Penrose introduced his “Law”, according to which the size of the prison population is inversely related to the available number of psychiatric hospital beds [23]. Some studies have indeed found a correlation between the decrease in psychiatric bed numbers and an increase in mentally ill prisoners [24] but further research is needed to establish causality [25, 26]. It has been suggested, for instance, that other societal factors in the USA and Western Europe, rather than deinstitutionalization in itself, are responsible for the rising number of prisoners with psychiatric problems. Significant contributory factors may be the increase in homelessness, illegal drug use, changes in mental healthcare funding [24], and decreased numbers of forensic psychiatric pre-sentence examinations [27]. Furthermore, a cross-sectional study by Large and Nielssen [28] found no association between the size of the prison population and psychiatric hospital bed numbers in high-income countries, whereas positive correlations were found in low- and middle-income countries.

However, while ongoing trends in psychiatric service systems indeed stress deinstitutionalization and out-patient care, for some patients a restricted and safe environment is an essential feature of their mental health care regime, at least at certain stages of their illness, in order to maintain both their own and public safety. Here, we review the current literature on what aspects of forensic psychiatric facilities best serve the needs of those patients who still need to rely on them, and we attempt to define a structured way of approaching the complex and costly challenge of forensic psychiatric facility design. For the purpose of this review, we define a forensic psychiatric facility as a healthcare institution into which patients have been diverted from either correctional services, typically due to criminal irresponsibility issues or enduring post-sentencing mental illness, or general psychiatric services, typically due to serious risk of inter- or intrapersonal violence.

## General remarks on psychiatric hospital design: history, ideology, evidence

According to Horsburgh [29], the architectural design of psychiatric facilities and the quality of living space are essential aspects of the healing process, and thus affect the outcome of medical care. Previous studies have highlighted the importance of a safe physical environment that enables intensive, stabilizing treatment together with reasonable privacy and observability [30]. Dijkstra, Pieterse [31] conclude that while the general notion of the healthcare environment affecting the well-being of patients is supported by the literature, conclusive evidence is still limited as to specific environmental factors. Others argue that research findings and clinical conjecture have consistently supported the idea of the environment playing a significant role in patient and staff functioning in psychiatric hospital settings, but that this fact has even yet to be fully recognized and implemented in hospital design. Indeed, the development of psychiatric institutions has been characterised as coercive, confining and their buildings as the embodyment of discipline and control, the exercise of power over the socially deviant [32]. Yet modern design of built environments can create an engineered sense of shared safety and ownership of one’s living space [33, 34] and even the earliest hospitals for the mentally ill were established with caring and compassionate ideals [35]. Typically, the various designers and theorists placed great emphasis on the same elements of architecture, staffing and activities: physical, relational and procedural safety and security as described below, with an organisational and managerial vision and overview [36, 37]. In what may be a series of historical cycles, these elements became subsequently victims of the social processes that supported them [38]: a growing awareness of the social context of mental illness [39] led on to a recognition of the anthropological micro-cultures of closed institutions [40, 41] and the policy decision to close the asylums on the grounds that they were inherently harmful and community care would prevent ‘institutionalisation’ followed [42]. In practice, it was evident from an early stage that this was not a coherent theory or policy, and the practice of decarceration was not equal to a liberation or an improvement in quality of life [19, 43]. As the historical cycle has continued to turn, there has been first a new interest in reform and attention to therapeutic environment and regime [44, 45], a review of the culture and exercise of ‘power and praxis’ in psychiatric wards and hospitals [46] and a late, but welcome, interest in the primary therapeutic power of the hospital environment itself [29, 31, 47–52], in which nature and art play an important part [53].

All in all, one of the most consistent recommendations in the body of literature on psychiatric hospital design is to reduce the institutional feel of the facility, and instead create a more homelike environment [54]. This must be taken into consideration from the earliest stages of the design process, as architectural features, such as the layout or physical plan, size and shape of rooms, and the placement of windows, are relatively immutable aspects of the hospital building [50]. In contrast, interior design features are less fixed; furnishings, basic consumer appliances (e.g. televisions, telephones, computers), colors, artwork and the décor of patient rooms [50] are easily modifiable according to purpose and individual choice. Also ambient features, such as sunlight, views of nature and reduction in noise, have been shown to be helpful in psychiatric recovery [31, 55]. Also, fresh air, good ventilation, neutral odours, and natural daylight in patient rooms can promote the recovery of psychiatric patients with severe depression; in general, soft, indirect and pervasive lighting should be provided [54]. Providing outdoor gardens and rooms with views of nature might shorten recovery times, reduce need for pain medication, and mitigate patients’ psychological distress by serving as positive distractions [47, 54]. These findings are consistent with research indicating that views of nature produce higher levels of relaxation as compared with urban scenes [48]. According to Edgerton, Ritchie [33], colour selection, live plants, floor space, and furniture arrangement can foster social interaction, but at the same time help maintain privacy. This is an important point, as patients should be able to regulate their own level of social contact while in psychiatric care [52, 54].

Thus, although the trend towards deinstitutionalizing psychiatric patients remains strong, the modern psychiatric unit continues to have an important role to play as a stable and secure centre for treatment, research, and rehabilitation [51]. This is particularly true of forensic psychiatric units.

## Forensic facilities: therapy integrated with security

In the UK, as an example, the current forensic psychiatric services began to form and diverge from earlier settings in the early 1960s. Regional secure units were set up within general psychiatric hospitals from the 1980s onwards, further stimulating debate about how health and social services should provide for mentally ill offenders and non-offenders with similar needs. The Reed Report [56] made hundreds of recommendations for streamlining forensic service provision, including that security measures should be no stricter than warranted by the risk posed by patients to themselves or to others. Accordingly, Eggert, Kelly [49] emphasize that the cardinal principle in the design of psychiatric facilities is the mitigation of the risk of self-harm or harm to others. Nowhere is this more true than in forensic psychiatric facilities.

Indeed, risk issues lie at the very core of forensic psychiatric practice, as the environment must be safe and stable before any real treatment progress can take place [57]. In a forensic facility, various forms of treatment- and safety-compromising risks are present which require correlative management strategies. For instance, the risk of violence and self-harm requires adequate numbers of well-trained clinical staff [58] and a culture of fluent communication of risk observation [59]. Also, the treatment facilities must be designed in a way that enables staff to observe all activity that occurs in the interior areas, except, for reasons of privacy, the patients’ rooms [30], which in turn must be designed to minimize ligature risk [60]. The risk of escape, on the other hand, requires a well designed perimeter and well thought-out procedures regarding passage in and out of the secure facility [2]. An unsuitable physical environment can severely impair care quality and the feeling of safety, and might damage the person-centered therapeutic process [45, 61].

### The three aspects of security

In order to overcome the risk-related challenges set out above, the Reed Report distinguishes three aspects of security: physical security, relational security and procedural security [56]. Physical security covers aspects of environmental and building design that include safety and restraint, such as safety-windows, locks, walls and alarm systems. Relational security focuses on a more qualitative viewpoint: the patient–professional relationship, knowledge of specific patients’ history and a general understanding of the forensic patient population. Procedural security focuses on policies and procedures that maintain safety and security, e.g. search protocols and surveillance of restricted items [56, 59, 62].

According to Kennedy [63], relational security is by far the most important element in maintaining a therapeutically safe and secure setting and furthering of patients’ therapeutic progress. However, the physical environment affects the way relational security is delivered [59]. Thus, security measures and therapeutic issues are closely linked; neither should be dealt with in isolation. Security provides a positive and supportive framework within which clinical care and therapy are safely delivered. Good security and effective therapy should be seen as integrated concepts rather than opposite ends of a spectrum, keeping in mind that secure psychiatric facilities are distinct and separate from prisons; the building and site layout must be planned in a sensitive and balanced way. Thus, a therapeutic forensic hospital milieu must include the therapeutic use of security, enabling a better quality of life through effective, individualized medical interventions and rehabilitative social interaction, such as multidisciplinary teamwork, occupational therapy and other meaningful activities [15].

With these factors in mind, the UK Department of Health has set out binding principles and security requirements for designing secure inpatient settings in Great Britain in the Environmental Design Guide [4]. A safe and therapeutic environment, which is fit for purpose and takes into account that patients may be in residence for extended periods, is a key precondition for relational security to develop. Thus, the Environmental Design Guide lays out meticulous instructions for the planning and design of individual 15 m^2^ en-suite bedrooms, interview or consulting rooms for one-to-one and group activities, visiting areas, day rooms, and therapeutic areas for occupational therapy, sports and exercise. The guide states that security and safety for patients, staff and public must always be taken into consideration while designing and building windows, doors, walls and fences. Materials accessible to patients should be non-breakable. Windows must provide daylight and ventilation, but at the same time maintain security, and be resistant to scratches and damage. Doors and entrances, electronic systems for personal security, internal walls and floor surfaces, ceilings, lighting, corridors, pitched roofs, fire precautions and fences along the external perimeters all have their own specifications. The guide also recommends incorporating a de-escalation area into the site design—a low-stimulus environment for patients to calm down, which acts as a step between seclusion facilities and the ward area. Bathrooms and lavatories should be designed to reduce the risk of suicide; thus, all ductwork, plumbing and pipe-work should be concealed. The guide also includes testing schedules for building materials and information about the installation and use of CCTV. For staff, it recommends the provision of spaces for confidential working, learning and development, eating and resting, as well as changing rooms and locker facilities [4].

### Urban vs. rural setting

Various historical and geographical factors have contributed to the fact that some countries have secure units located in densely populated urban areas, whereas in others, forensic facilities are more often situated in remote locations.

Urban forensic services can provide various forms of rehabilitative stimuli which are not so easily accessible in a more rural environment. But this in turn raises issues concerning the safety of both the patients themselves and people around them: access to drugs and alcohol, and opportunities for antisocial interaction are all factors to be taken into consideration. Regardless of the setting, buildings must be secure, so as to facilitate the treatment model and care pathway, and simultaneously to promote community engagement and recovery. Using high-quality construction materials throughout the building, and being mindful of how operational and structural aspects of the hospital building integrate into its environment, will help to improve outcomes for patients [4].

Proposals to build a forensic facility in an urban area tend to raise media attention, and opposition from local residents concerned about the risk of violence and criminal activity in their communities (As an example see [64]). However, it has been demonstrated that medium-security units do not have a measurable impact on serious crime rates in their immediate localities [65]. Accordingly, it is recommended to locate medium- and low-security units as close as possible to general population services, so as to enable patients to maintain contact with their family and friends, general psychiatric services and out-patient care, and the community at large [5]. Although intoxicants, criminality and conditions favourable to recidivism are less prevalent in areas with low population density [66], these risks must still be invariably factored in as potential key destabilizers in any setting. Thus, monitoring and intervening in antisocial activities, including the use and trading of drugs, are key requirements in any forensic service provision.

### Privacy vs. spaces for social interaction

According to Karlin and Zeiss [54], accommodating patients in single-occupancy rooms enhances their sense of autonomy and might promote participation in therapeutic activities. On the other hand, Dvoskin, Radomski [30] cited enhanced opportunities for social interaction with other patients and possible cost savings as advantages of multiple-occupancy rooms, but increased risk of violence due to forced co-habitation as a drawback. Also, having an integrated toilet and sink in every patient room has its benefits and drawbacks; the advantage of en suite rooms is that they are more convenient for patients and they make the nighttime corridor surveillance easier. On the other hand, en suite rooms are more expensive than “dry rooms”, and might be reminiscent of a prison environment. There are also concerns that spending too much time in their rooms might hinder patients’ rehabilitation [30]. En suite designs should take fully into account the risks posed by potentially destructive, polydipsic and suicidal patients; for example, water supply must be adjustable from outside patients’ rooms. However, all in all, recent recommendations favour en suite patient rooms [4, 6], as the benefits provided by privacy and the chance to personalize one’s immediate surroundings outweigh possible drawbacks.

### Seclusion and restraint

National mental health laws in most countries stipulate that psychiatric patients who exhibit dangerous and difficult-to-treat behaviors can be subjected to coercive measures, such as seclusion [67, 68], if specific criteria are met. However, being subjected to coercive measures can cause significant displeasure [61]—even when considered necessary by patients themselves [69]—and might be experienced as traumatic and unjust [70]. Naturally, it is preferable to prevent or reduce the risk of patients’ displaying the kind of behaviours that warrant coercive measures being deployed. Patients should therefore have access to quiet, conveniently located areas, where they can relax and aggression can de-escalate, such as secure garden spaces which patients can enter at will [61].

As for the location of seclusion rooms, placing them near to the center of activities might promote safety, whereas locating them closer to the periphery might soothe the ward environment but reduce the immediate availability of staff. Hence, seclusion rooms should be placed near nursing stations, but outside the main corridors and dayrooms [54]. Their construction should be highly robust, in order to withstand repeated destructive violence without posing a risk for the patient or staff, whilst at the same time maintaining the dignity and comfort of the secluded patient [4, 6, 71].

### Managing on-site substance misuse, escapes and absconding

Most forensic inpatients suffer from psychotic illness with comorbid personality disorder and/or substance misuse [65]. Alcohol and drug misuse aggravate the symptoms of mental illness, increase impulsivity and risky behavior, and reduce the efficacy of treatment [72]. Well-functioning physical and procedural security, such as fences, check-ups and staff observation, in addition to efficient relational security, are ways to moderate alcohol and drug misuse in a ward setting [73]. However, legal constraints may hinder the development of routine security protocols due to the various ways in which the concept of “blanket restriction”, i.e. the routine application of rules or policies that infringe upon a patient’s right to self-determination, without individual risk assessments to justify their application [74, 75], is defined in national legislation and interpreted by respective medico-legal authorities. On the other hand, forensic units are expected to maintain an appropriate level of security, and it has been shown that absconding is most likely to occur when patients themselves feel that wards are unsafe [76]. Also, an increase in bed numbers is associated with a rise in the incidence of escapes, as is—unsurprisingly—a lower perimeter fence [77].

### Human rights

According to Gradillas, Williams [65], the development of secure forensic inpatient facilities is vital to the provision of humane and effective treatment of the mentally ill in any society. Accordingly, in recent years, human rights have become an increasingly pivotal focus of public debate. People with mental illness, who pose a risk to themselves and to others, are some of the most vulnerable members of society, and special attention must be paid to protecting their rights [78]. The mentally ill offender has a dual position: as a patient in need of treatment and as a person subject to the criminal justice system. In many European countries, continual attempts are being made to create a better legal balance by ensuring the right to adequate treatment for all individuals, while at the same time maintaining public safety [14]. Although the clinical challenges that forensic services face recur across different countries, variability in national legislation causes significant differences at the service-patient interface, even in countries that have ratified and subscribe to the same international human rights legislation and are subject to the same monitoring bodies [68].

A central concept that arises in any consideration of these issues is that of the “inherent dignity” that the Universal Declaration of Human Rights [79] ascribes to all persons [80]. The ways that inherent human dignity can be upheld by mental health services can generally be approached in two ways: by defining dignity as empowerment, or as constraint. The first conceptualization defines dignity as a right to self-determination, rather than having limits placed on one’s free choices. The latter, on the other hand, defines dignity as an objective value that reaches beyond the free choices of an individual; thus, a person’s dignity can be compromised by his own actions regardless of whether the individual chose to act as he did at the given time [81]. Also, constraining one individual can be seen as maintaining the dignity of another, if the latter is somehow threatened or, indeed, damaged by the actions of the former. As these concepts of dignity are obviously not mutually exclusive, an ideal balance—although difficult to attain—must be striven for when planning all aspects of forensic services, from building design to service policies and clinical treatment measures.

## Template for a forensic facility design brief

Taking into consideration the myriad clinical, legal and architectural considerations, planning a new forensic hospital is a highly complex process. However, as an integral part of a well-functioning forensic service, it is a clinical and ethical necessity in any society. Despite variability in national legislation and socio-demographic factors affecting various national forensic patient populations, in the following design template we attempt to capture general, internationally applicable design principles (Table 1).

**Table 1:** Template for a forensic facility design brief

Main design issues to be addressed	Details	Tasks and tools
*1. Define the need*		
Define the patients that are to be served by the facility	What clinical and legal issues affect patient selection; i.e. who are the forensic inpatients to be catered for? What variation is there in the patients’ length of stay? What is the diagnostic variation in terms of psychotic states, personality disorders, substance abuse, autism spectrum disorders, learning disabilities, paraphilias, mood disorders, organic brain disorders and somatic problems? What is the patient profile in terms of gender, age, ethnicity, risk level, and acuteness of symptoms?	Compile demographic, legal and clinical descriptors to be used to define and measure patient characteristics relevant to clinical and security needs 1. Demographic and legal descriptors [14]. See also WHO AIMS 2.2 [82] 2. Patient characteristics as per DUNDRUM-1 [83] and DUNDRUM-2 [84] 3. Diagnostic data according to ICD/DSM 4. Define risk-level criteria by using suitable structured clinical judgement-tools (e.g. HCR-20 [85], START)
What is the facility’s role and profile in the overall organization of forensic services, general psychiatric services and prison services?	Which service should, or is legally bound to, care for each patient category?	1. Map existing assets and resources (institutions, buildings, staff, skills) [63] 2. Map existing pathways, legal processes and clinical criteria (e.g. DUNDRUM-3, DUNDRUM-4) [86] 3. Map existing volumes of flow (admissions, transfers and discharges) between the parts of the map
Does the facility have a role as a university-affiliated research and teaching hospital?	How must academic needs be catered for? How can the facility’s status and recruitment-appeal be raised by high-quality teaching and research?	1. Define training needs in order to establish and maintain care quality [87] 2. What research is required to fulfil the facility’s tasks?
*2. What are the options?*		
What issues in the service have created the need for a new facility?	How well are current facilities serving the patients in terms of treatment and security? What can be done do develop the service without building new facilities?	Compare possibilities against each other and against the ‘do nothing’ option by defining a set of scoring criteria and a scoring grid
Can existing facilities be renovated/upgraded?	Can existing buildings be modified with reasonable cost and effort, as opposed to designing a completely new facility?	
What options are there for locations?	Urban vs. rural? What synergy can be obtained from other nearby services? How can accessibility be facilitated for families of patients, and visiting professionals? How can access to courts, and prison and hospital transfers etc. be most conveniently managed? Where is the best-quality staff located? What location has recruitment appeal? How will the neighbours react?	
*3. Policy issues*		
Regional policy	What is the attitude of regional medico-legal authorities, healthcare organizations and other services, such as the police and social services? What level of knowledge and support is available from these bodies?	National and international policy makers, medico-legal authorities, service users, their families/carers and staff should all be engaged in each stage of the development and implementation process [68, 88]
National policy	What is the relevant national legislation? Does current legislation support your facility development plans? If not, can legislation be changed? What is the correct level for creating a supportive and healthy discourse on forensic issues among national decision-makers?	
International policy	Are the developmental plans in line with international recommendations, such as those formulated by relevant professional bodies and quality networks, CPT and WHO?	
Financial policy	What are the financial resources available to invest in the development project? Which collaborators and national and international funds must be explored as possible sources for funding?	
Patient advocacy	Are the development plans in line with the policies of patient advocacy organizations?	
*4. Functional content*		
Quality of design and materials	How can therapeutic and security requirements be combined in the design? How are the different facets of security, i.e. physical, procedural/organizational and relational, taken into consideration? How is the way patients are valued evidenced in the quality of materials? How are the materials tested in terms of being subject to unusual force and other stress? How are life-space issues taken into consideration, particularly the need to prevent institutionalization and boredom?	See above. Monitoring bodies, service users, carers and staff should be involved at each stage by establishing groups to participate in the process [4]
How will different needs be catered for?	How will the overall lay-out of the facility be structurally compartmentalized to serve patient populations, as defined in 1 and 5, with different needs? How will the overall design support rehabilitative progress through the facility? What is the pace/rhythm of treatment interventions in different subsections of the facility? What range of professional skills is needed? What number of staff must be recruited in each professional category?	1. Define TAU (treatment as usual) [89] 2. Define programs necessary to deliver TAU 3. Draft space and adjacency requirements to enable provision of all the necessary clinical services and supporting services
What services can be outsourced or linked with surrounding facilities and services?	E.g. pharmacy, kitchen, cleaning, maintenance, IT-services, personnel services	
*5. Growth and change*		
What issues must be taken into consideration in order to ensure that designs have longevity and don’t become outdated too soon?	Design to accommodate high security, and grade down if clinically possible Design for flexibility of patient transfers, both within the facility and in and out of it Take into consideration the change in demographics in society at large, e.g. aging of the population, immigration, growth of urban areas, possible social decline in catchment area, substance misuse trends Take into consideration that if the number of general psychiatric beds fall**s**, for instance due to national policy, there may be an increase in pressure on the remaining (forensic) beds	Again, planning ahead means involving the various experts and interest groups mentioned above in each step of the planning process

## Discussion

The process of deinstitutionalization of psychiatric care in many Western nations is coming to be understood, rather, as a more complex process of trans- or reinstitutionalization from traditional psychiatric hospitals to supported housing, prisons and, not least, forensic psychiatric units [19, 22, 27]. As a result, authorities in many countries have come under increased pressure to both expand and update their forensic psychiatric services [17, 90–93], so as to respond more effectively to the needs of mentally ill offenders and other psychiatric patients with similar needs. Simultaneously, an increased focus on patient and carer rights and involvement has emerged throughout the development and implementation of healthcare services [94, 95], as have international monitoring standards to uphold human rights within psychiatric institutions [68]. Thus, it is becoming less acceptable to operate national forensic services without reference to international standards or without knowing what practices are employed elsewhere in the world [11, 96].

Developments in international trends, societal attitudes towards mental health issues, and the design of psychiatric care environments are closely intertwined. This is reflected in modern evidence-based architectural hospital design, which is founded on research into the link between characteristics of the care environment and patient recuperation and well-being [97]. Also, the way in which society defines its basic ethical principles changes over time, and it is a matter of continual ethical, clinical and legal debate where the line between security and therapy should be drawn at any given time. Thus, changing views on treatment must be matched by changes in the operational models of healthcare services, which are themselves bound up with the design of hospital buildings [51].

## Conclusions

Forensic psychiatric hospitals are high-cost, low-volume medical institutions, which are required to provide both therapeutic and safe and secure material environments [2, 4, 6, 59, 68]; thus they are at the very forefront in implementing the latest developments in medical architecture. Although the evidence base for many interventions within forensic psychiatry is still weak, these hospitals do provide an essential, specialized service for mentally disordered offenders and others with similar needs, both in terms of healthcare and decreasing the risk of re-offending [15, 98]. Only by entering into an open, yet structured and coherent, international research-driven discussion with clinicians, scientists, architects, policy-makers and medico-legal authorities, can we continue to raise the standard of our services, and the facilities in which they are provided.
